# A plea for multilateralism

**DOI:** 10.1007/s11098-026-02481-6

**Published:** 2026-02-20

**Authors:** Luca Incurvati

**Affiliations:** University of Amsterdam, Amsterdam, Netherlands

## Abstract

In their terrific book *Reasons for Logic, Logic for Reasons*, Ulf Hlobil and Robert Brandom defend a normative-pragmatic interpretation of implication and incompatibility cashed out in bilateral terms. Using epistemic modal cases, I argue that Hlobil and Brandom’s normative-pragmatic interpretation of implication fails to account for the force of consequence and to provide an extensionally adequate characterization of implication. I also show that the same cases cause trouble for Hlobil and Brandom’s condition relating negation to incompatibility. I argue, however, that these problems can be avoided by switching from a bilateral to a multilateral setting.

## Introduction

Ulf Hlobil and Robert Brandom have written a terrific book. *Reasons for Logic, Logic for Reasons* (henceforth RLLR) is a major contribution to the inferentialist programme and, more generally, to the philosophy of language and the philosophy of logic. And it also contains a wealth of novel technical material which will be of interest to logicians and formal semanticists working in the proof-theoretic and model-theoretic traditions alike.

Technically, Hlobil and Brandom give us a proof theory (with various extensions and modifications), as well as not one but two model theories. The first is a version of Kit Fine’s (2017a; 2017b) truth-maker semantics generalized to allow for the failure of various structural rules. The second is implication-space semantics, whose original version is due to Brandom’s student Dan Kaplan and which generalizes Jean-Yves Girard’s phase-space semantics for linear logic.

Philosophically, Hlobil and Brandom defend several key theses: (i) contents are to be explained in terms of reason relations (*semantic inferentialism*); (ii) reasons relations are neither monotone nor transitive (they are *open*, as Hlobil and Brandom put it); (iii) reason relations should be understood bilaterally, as reasons *for* (implication) and reasons *against* (incompatibility); (iv) reason relations can be explained in a normative-pragmatic fashion (in terms of what combinations of assertions and denials of sentences are normatively possible) or in an alethic-representational fashion (in terms of what combinations of truth makers and false makers for the worldly propositions of sentences are alethically possible); (iv) the normative-pragmatic explanation and the alethic-representational explanation can be reconciled, in that they are concerned with different aspects of the same phenomenon, *viz.* the structure of reason relations; (v) this structure itself is best explicated in more abstract terms, by means of implication-space semantics, which is an intrinsic meta-vocabulary (unlike the normative-pragmatic and alethic-representational meta-vocabularies, it does not appeal to conceptual resources beyond those of the relevant base vocabulary); (vi) the structure of reasons can also be explicated via another intrinsic vocabulary, namely logical vocabulary, which is not wholly meta-linguistic: although it serves to talk about reason relations, it makes no use of overt meta-language; (vii) indeed, the function of logical vocabulary, and what distinguishes it from other vocabularies, is precisely that of making it possible to talk about reason relations in the very language whose reason relations are under discussion (*logical expressivism*).

Clearly, then, this is a very rich book, and I cannot hope to tackle even superficially all of the themes it covers. I will restrict attention to issues surrounding Hlobil and Brandom’s normative-pragmatic interpretation of implication and incompatibility and their account of the relation between incompatibility and negation. Using epistemic modal cases, I will argue that Hlobil and Brandom’s normative-pragmatic interpretation of implication fails to account for the force of consequence and to provide an extensionally adequate characterization of implication. And using the same cases, I will also show that Hlobil and Brandom’s condition relating negation to incompatibility is problematic. I will argue, however, that these problems can be avoided by switching from a bilateral to a multilateral setting.

## From normative pragmatism to bilateralism

It will be helpful to start by providing some historical background. The publication of *Making it Explicit* (Brandom, 1994) was of momentous importance in the development of inferentialist semantics. Among many other things, Brandom argued that we could explicate our discursive practice in terms of the notions of commitment and entitlement. Notably, he used commitment and entitlement to characterize several semantic notions (Brandom, 1994, pp. 168–169): $C$ is a *committive consequence* of $A_1, \dots , A_n$ just in case anyone committed to $A_1, \dots , A_n$ is thereby committed to $C$; $C$ is a *permissive consequence* of $A_1, \dots , A_n$ just in case anyone committed and entitled to $A_1, \dots , A_n$ is thereby (*prima facie*) entitled to $C$;^1^ and $A$ is *incompatible* with $B$ just in case commitment to $A$ precludes entitlement to $B$.

This conceptual apparatus was not formally implemented. Cutting a long story short, Brandom, under the influence of Hlobil and Kaplan, eventually settled for a multiple-conclusion sequent calculus as the logical framework for his normative pragmatism. Technically, the sequent calculus offers the advantage of providing a particularly friendly home for open reason relations, an advantage on which Hlobil and Brandom cash in. But the use of multiple conclusions (which ensures the classicality of the purely logical part of the calculus) also comes with a familiar challenge: examples of multiple-conclusion arguments in the wild are seemingly nowhere to be found; but then, in what sense does a multiple-conclusion system provide a model of how the inferences that we draw in our actual linguistic usage determine meaning?

Florian Steinberger (2011) provided the most lucid formulation of this challenge, and Hlobil and Brandom elect to meet it by endorsing the bilateral interpretation of the sequent calculus pioneered by Greg Restall (2005) and further developed by Ellie Ripley (2013). This is, specifically, an interpretation of the implication/consequence relation holding between the premisses and the conclusions of a sequent. According to this interpretation, a sequent $\Gamma \succ \Delta$ is to be read as saying that it is incoherent (‘out of bounds’) to assert all members of $\Gamma$ and deny all members of $\Delta$, where the speech acts of assertion and denial express, respectively, the practical attitudes of acceptance and rejection. In the deontic normative vocabulary adopted by Hlobil and Brandom, the bilateral interpretation of implication takes the following form, suggested by Ryan Simonelli:^2^

**[Implication]**

$\Gamma \succ \Delta$ ($\Gamma$ implies/is a reason for $\Delta$) if and only if commitment to accept all members of $\Gamma$ precludes entitlement to reject all members of $\Delta$.

Incompatibility is then characterized as follows:

**[Incompatibility]**

$\Gamma \sharp \Delta$ ($\Gamma$ is incompatible with/is a reason against $\Delta$) if and only if commitment to accept all members of $\Gamma$ precludes entitlement to accept all members of $\Delta$.

In a footnote (p. 155, fn. 7), Hlobil and Brandom take the bilateral interpretation as ‘offering a natural connection between our use of sentences and consequence relations’ and hence as putting Steinberger’s worry ‘that semantic inferentialism is in tension with multiple-conclusion calculi’ to rest.

## On a purported reduction of committive consequence

Steinberger had already considered the bilateral interpretation in his paper and had found it wanting from an inferentialist point of view. Steinberger’s point is that, on the bilateral interpretation, all we are told is how the meanings of the logical constants *constrain* what can be normatively asserted and denied.^3^ For instance, Hlobil and Brandom’s left rule for conjunction (together with the fact that the rule is invertible) tells us that it is normatively impossible to accept $A$ and $B$ together with all of the sentences in $\Gamma$ while rejecting all of the sentences in $\Delta$ just in case it is normatively impossible to accept $A \wedge B$ together with all of the sentences in $\Gamma$ while rejecting all of the sentences in $\Delta$. But this does not allow us to determine the conditions under which one is committed to accepting a conjunction. Similarly, the sequent calculus rules for conjunction do not allow us to determine the commitments of someone who has committed to accepting a conjunction.

From a normative-pragmatic perspective, the diagnosis should be clear. The bilateral interpretation does not afford us the means to characterize committive consequence. Or so it would seem. Hlobil and Brandom (p. 47) subscribe to the following principles (again suggested by Simonelli):

**[Pragmatic Implicit Acceptance (PIA)]**

Any set of commitments that precludes entitlement to reject $A$ implicitly commits one to accept $A$.

**[Pragmatic Implicit Rejection (PIR)]**

Any set of commitments that precludes entitlement to accept $A$ implicitly commits one to reject $A$.

We have, in effect, a reduction of committive consequence (now understood bilaterally, so that commitments are not commitments to sentences but to acceptances or rejections of sentences) to the bilateral interpretation of implication and incompatibility. I want to make two observations about this proposed reduction.

The first observation is that even if the reduction works, it only works for single conclusions. We cannot generalize PIA by saying that if commitment to accept all of $\Gamma$ precludes entitlement to reject all of $\Delta$, then it thereby implicitly commits one to accept one of $\Delta$. Consider, for instance, the sequent $A \vee B \succ A, B$. We cannot say that if commitment to accept $A \vee B$ precludes commitment to reject $A$ and to reject $B$, then commitment to accept $A \vee B$ implicitly commits one to accept $A$ or accept $B$. One can be committed to accept a disjunction without being committed to accept either of the disjuncts.^4^

The problem is avoided, of course, if we generalize PIA by saying instead that if commitment to accept all of $\Gamma$ precludes entitlement to reject all of $\Delta$, then it thereby implicitly commits one to accept the disjunction of all members of $\Delta$. But this is tantamount to falling back on the disjunctive reading of multiple conclusions, which is precisely what the bilateral interpretation of multiple-conclusion calculi was designed to avoid.

Our predicament is familiar: when we try to explain how multiple-conclusion calculi can account for the committive import of implication—what Ian Rumfitt (2000) calls the ‘force of consequence’—we seem to be forced to treat their conclusions not as genuine conclusions, but as the disjuncts of a single disjunction, which acts as the real conclusion. As Steinberger (2011: 353) observes, it is significant that the point seems to be conceded by Restall himself:

once one reads this turnstile as a form of *consequence* from $X$ to $Y$, one must read $X$ and $Y$ differently—it is the *conjunction* of all $X$ that entails the *disjunction* of all $Y$ (Restall 2005: 8, fn. 3, emphases in the original).

As a matter of fact, Hlobil and Brandom only consider the reduction of committive consequence to the bilateral interpretation when considering single-conclusion reason relations. But in order to rehabilitate multiple-conclusion calculi as accounts of implication what needs explaining is how they can account for the force of consequence in the case in which multiple conclusions are countenanced. This is especially important given Hlobil and Brandom’s concession (p. 132) that there are areas of enquiry, such as mathematics and physics, in which reason relations (including, presumably, implication) obey classical logic. For Hlobil and Brandom purport to give this point its due by showing that, when the problematic structural rules hold, the implication relation of their calculus is (supra-)classical, a result which rests on the calculus admitting of multiple conclusions.^5^

The second observation I want to make about the reduction of committive sequence to the bilateral interpretation is that, as it stands, it does not work. Hlobil and Brandom motivate PIA as follows (p. 48):

The claim of PIA is that if commitment to accept precludes entitlement to reject $A$, then that same commitment to accept implicitly commits one to accept $A$. One option, rejecting $A$, has been ruled out. One could remain agnostic, neither accepting nor rejecting. But that’s not right. After all, one of the options has been ruled out. One cannot become entitled to reject $A$. The only option left standing, the only one available that one could potentially be entitled to is accepting $A$. By hypothesis, one has not yet explicitly done that. But that attitude of acceptance is implicit in the ruling out (as something one cannot be entitled to) of the only other option, in the sense that it is the only option left open. This is not the same as actually adopting the attitude, and that is what we mark by calling the commitment to accept “implicit,” by contrast to the actual, explicit adoption of it. It seems clear both that this is an intelligible pragmatic sense of “implicit commitment to accept” and that calling it that is motivated by the rendering impermissible of the only other active option, rejection, and the consequent relative pointlessness of remaining uncommitted.

I have no qualms with Hlobil and Brandom’s talk of implicit commitment. I agree it is intelligible and, indeed, I think it is necessary to make sense of committive consequence, which can be understood as computing the commitments implicitly undertaken by a speaker given their explicit commitments (Incurvati and Schlöder 2023b, pp. 71–72). What I find problematic about Hlobil and Brandom’s argument for PIA is rather its assumption that entitlement to accept and entitlement to reject are the only two options. In particular, someone whose extant commitments preclude them from being entitled to reject $A$ may nonetheless be entitled to refrain from accepting $A$. All that follows is that they are committed to refrain from rejecting $A$. This is compatible with either accepting $A$ or refraining from accepting $A$. In the latter case, the subject would be agnostic towards $A$.

Here, to stress, it is crucial that to be agnostic about $A$ is not, as Hlobil and Brandom appear to suggest, to neither accept nor reject $A$. For this does indeed suggest that entitlement to accept and entitlement to reject are the only two options. Rather, as a longstanding tradition forcefully defended by Jane Friedman (2013, 2017) has it, agnosticism is best conceived as a genuine attitude. For one thing, as Friedman notes, we do not want to say that cave dwellers were agnostic about the existence of the Higgs boson simply because they neither accepted nor rejected its existence. The agnostic attitude, as Filippo Ferrari and I have argued, may be understood as the attitude one has towards a certain content if one *refrains from* accepting and rejecting that content (Ferrari & Incurvati, 2022). Thus, someone whose commitments preclude entitlement to reject $A$ but nonetheless refuses to accept $A$ need not be saddled with ‘the consequent relative pointlessness of remaining uncommitted’. Rather, they might prefer to commit to being agnostic about $A$. Indeed, the idea that it is possible to commit to being agnostic seems needed to make sense of certain types of disagreement. Consider a religious agnostic who thinks that there can never be evidence for or against the existence of God. On the face of it, an agnostic of this kind would seem to disagree with both the theist and the atheist. But if disagreement is to be modelled on the basis of incompatible commitments (as I think it should, and I suspect Hlobil and Brandom might agree), then, if the agnostic simply lacks commitments, there is no room left for disagreement (see also Ferrari 2022).

In a footnote (p. 54, fn. 2), Hlobil and Brandom appear to somehow qualify the scope of their argument for PIA, by noting that in order to provide a motivation for this principle, they are suppressing ‘many considerations that would be relevant in certain argumentative contexts’. They explicitly mention paradoxical contexts, and indeed there are good reasons to think that one ought to both refrain from accepting and refrain from rejecting the Liar sentence (Incurvati & Schlöder, 2023a). However, it seems clear to me that there are frequent and much more mundane contexts in which someone whose extant commitments preclude them from being entitled to reject a sentence ought nonetheless to refrain from accepting it.

Suppose, for instance, that having just woken up and with the blinds still down, Andrea tells her partner *It might be raining outside*, on the basis of the forecast she had read the day before, which predicted slight chances of rain in the morning. Andrea’s utterance explicitly commits her to accept *It might be raining outside*, which precludes entitlement to reject *It is raining outside*. It would be wrong to conclude, however, that Andrea is thereby committed to accept *It is raining outside*. Andrea may—and indeed, in the described scenario, is naturally taken to be—agnostic about whether it is raining outside.

Similar considerations apply to PIR. Consider a modification of the above scenario in which what Andrea tells her partner is *It might be that it is not raining outside*. This utterance explicitly commits Andrea to accept *It might be that it is not raining outside*, which appears to preclude entitlement to accept *It is raining outside*. But it does not follow that Andrea is committed to reject *It is raining outside*. What it does follow is that Andrea is committed to refrain from accepting *It is raining outside.*

These are the kind of cases that Julian Schloeder and I have used in Incurvati Schlöder 2017; 2019; 2023b to motivate countenancing further speech acts beyond assertion and denial, expressing the attitudes of refraining from rejecting (call it *weak acceptance*) and refraining from accepting (call it *weak rejection*).^6^ In our multilateral framework, PIA and PIR fail. But we do have that if someone’s extant commitments preclude entitlement to reject $A$, then they are implicitly committed to *weakly* accept $A$, and that if someone’s commitment to accept $A$ preclude entitlement to strongly accept $B$, then they are implicitly committed to *weakly* reject $B$. This is in line with the scenarios above. In the first scenario, Andrea’s explicit commitment to accept that it might be raining outside implicitly commits her to refrain from rejecting that it might be raining outside. In the second scenario, Andrea’s explicit commitment to accept that it might be that it is not raining outside implicitly commits her to refrain from accepting that it is not raining outside.

## Multilateralism to the rescue

The kind of cases I have considered raise deeper troubles. Recall that according to Hlobil and Brandom’s bilateral interpretation of implication, $\Gamma$ implies $\Delta$ if and only if commitment to accept all of $\Gamma$ precludes entitlement to reject all of $\Delta$. And in the first scenario above, Andrea’s commitment to accept *It might be raining outside* precludes entitlement to reject *It is raining outside*. It does not follow, however, that *It might be raining outside* implies *It is raining outside*. This would be an unacceptable modal collapse. This point does not only affect situations involving modal vocabulary. Reformulating an observation made by Steinberger (2011, p. 353) (who credits Rumfitt) in the normative-pragmatic vocabulary used by Hlobil and Brandom, it would seem that, just like the classicist, the intuitionist is precluded entitlement to (strongly) reject the Law of Excluded Middle ($\textsf{LEM}$). But it would of course be wrong to conclude that $\textsf{LEM}$ is an intuitionistic theorem.

No analogous issue is faced by Hlobil and Brandom’s normative-pragmatic characterization of incompatibility. Consider, for instance, the second scenario involving Andrea. Andrea’s acceptance of *It might be that it is not raining outside* precludes entitlement to accept *It is raining outside*. By the normative-pragmatic characterization of incompatibility, it follows that the two sentences are incompatible, as indeed they are.

This suggests that while Hlobil and Brandom’s version of the bilateral interpretation fails to characterize implication, it succeeds in characterizing incompatibility, which was Brandom’s main target when using the notion of preclusion of entitlement.^7^ But it also suggests, once again, that what is problematic in Hlobil and Brandom’s characterization of implication is not so much the use of preclusion of entitlement, but its use of a notion of strong rejection. For suppose we take the rejection in the characterization of implication to be weak, so that the notion of implication characterized is accordingly strong:

**[Strong Implication]**

$\Gamma$ strongly implies $\Delta$ if and only if commitment to accept all members of $\Gamma$ precludes entitlement to weakly reject all members of $\Delta$.

Then the problems disappear, since Andrea’s commitment to accept *It might be raining outside* precludes entitlement to (strongly) reject *It is raining outside* but not to weakly reject it. Indeed, as we saw, Andrea is naturally seen as refraining from both accepting and rejecting *It is raining outside*. Similarly, the intuitionist is not precluded entitlement to weakly reject $\textsf{LEM}$. Indeed, refraining from accepting appears to be an apt characterization of the intuitionist stance with respect to $\textsf{LEM}$. 

However, the suggestion of replacing Implication with Strong Implication should be taken with care. For, if taken on board unrestrictedly, it creates a problem for Hlobil and Brandom’s right rules for negation and the conditional. Consider the former, for instance:
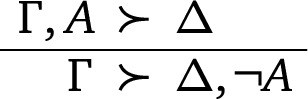


On the bilateral interpretation provided by Strong Implication, this says that if commitment to accept all of $\Gamma$ and $A$ precludes entitlement to weakly reject all of $\Delta$, then commitment to accept all of $\Gamma$ precludes entitlement to weakly reject all of $\Delta$ and $\lnot A$. But this does not follow. Let $A$ be *It is raining outside*, $\Gamma$ be empty and $\Delta$’s sole member be $A$ itself. Obviously, commitment to accept $A$ precludes entitlement to weakly reject $A$. It does not follow, however, that entitlement to weakly reject both $A$ and $\lnot A$ is precluded: we could be in a situation in which I am unsure about the weather and so am entitled to weakly reject both *It is raining outside* and *It is not raining outside*. When we characterize implication in terms of strong rejection, the problem disappears. For commitment to accept $A$ precludes entitlement to also *strongly* reject $A$. But it does follow from this that entitlement to strongly reject both $A$ and $\lnot A$ is precluded.

The conclusion I am inclined to draw is that Hlobil and Brandom should become multilateralists and countenance both weak and strong forms of rejection. One approach would be to have sequents to be read according to Implication as well as sequents to be read according to Strong Implication. This approach would lead, I suspect, to the kind of multilateralism recently advanced by Heinrich Wansing and Sara Ayhan (2023). Another approach would be to use signed sentences, that is sentences decorated by signs standing for speech acts or attitudes, such as assertion (acceptance) and weak and strong forms of denial (rejection). This is the approach adopted by Schloeder and myself in a natural deduction setting and by Simonelli (forthcoming) in a single-conclusion sequent setting.^8^

One advantage of the many-sign approach over the many-sequent approach is that it allows us to make immediate sense of of committive consequence, and in a very general way. In the system of Incurvati and Schlöder 2023b, for instance, we have a coordination principle (that is, a principle governing the interaction of speech acts and the attitude they express) which codifies the fact that if one’s extant attitudinal commitments (represented by the set of signed sentences $\Gamma$, which need not contain only accepted sentences) preclude someone from being entitled to reject $A$ (in symbols: $-\hspace{-0.1em}A$), then they are committed to weakly accept $A$ (in symbols: $\oplus \hspace{-0.08em}A$): 
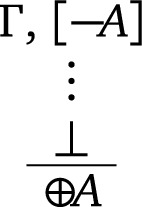


The scenarios involving Andrea and the weather also put pressure on a key principle relating incompatibility and negation to which Hlobil and Brandom subscribe:

**[Incoherence-Incompatibility Condition on Negation]**

$\Gamma$ implies $\lnot A$ just in case $\Gamma$ is incompatible with $A$.

Consider again the scenario in which Andrea has undertaken the commitment to accept *It might be that it is not raining outside*. This commitment, we saw, precludes entitlement to accept *It is raining outside*. Given Hlobil and Brandom’s pragmatic-normative characterization of incompatibility, this means that *It might be that it is not raining outside* and *It is raining outside* are incompatible. By the Incoherence-Incompatibility Condition on Negation, however, it follows that *It might be that it is not raining outside* implies *It is not raining outside*, which is again an unacceptable modal collapse (Russell & Hawthorne, 2016). It is to avoid modal collapses of this kind that the unrestricted meta-rule of reductio fails in the epistemic multilateral logic developed in Incurvati and Schlöder 2019; 2022; 2023b.^9^ From the fact that someone’s extant attitudinal commitments are incompatible with acceptance of $A$ (in symbols: $+\hspace{-0.1em}A$), all that follows is that they are committed to weakly reject $A$ (in symbols: $\ominus \hspace{-0.08em}A$).
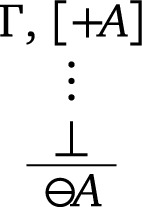


Hlobil and Brandom take the Incoherence-Incompatibility Condition to be central to their logical expressivism. In particular, they take this condition to ensure that negation can perform its expressive role as best as it can: ‘anything less counts as expressive impoverishment’ (p. 70). But what the scenarios show, in fact, is that the minimal incompatible of $A$—a sentence implied by everything that is materially incompatible with $A$—is not $A$’s negation but *It might be the case that not *$A$. And, using the second displayed coordination principle, one can derive (the assertion of) $\Diamond \lnot A$ in multilateral logic from any $\Gamma$ incompatible with (the assertion of) $A$.^10^

Is it possible to formulate a modified version of the Incoherence-Incompatibility Condition on Negation which avoids the foregoing problems? Yes, it is. What we need is a stronger notion of incompatibility:

**[Strong Incompatibility]**

$\Gamma$ is strongly incompatible with/is a strong reason against $\Delta$ if and only if commitment to accept all of $\Gamma$ precludes entitlement to weakly accept all of $\Delta$.

With this notion on board, we can then formulate the following condition on negation:

**[Strong Incoherence-Incompatibility Condition on Negation]**

$\Gamma$ implies $\lnot A$ just in case $\Gamma$ is strongly incompatible with $A$.

Clearly, although *It might be that it is not raining outside* is incompatible with *It is raining outside*, it is not strongly incompatible with it, since accepting *It might be that it is not raining outside* does not preclude entitlement to weakly accept *It is raining outside*. At the same time, *It is not raining outside* is strongly incompatible with *It is raining outside*, since accepting *It is not raining outside* does preclude entitlement to weakly accept *It is raining outside*. We see, therefore, that even the problems surrounding conditions that, by Hlobil and Brandom’s own lights, are central to their logical expressivism may be addressed by switching to a multilateral perspective. And again, the many-sign approach allows us to provide a more general point of view on the matter in a very straightforward way. For we have a coordination principle codifying the fact that if one’s extant attitudinal commitments (again represented by a set of signed sentences $\Gamma$, possibly including, but not necessarily limited to, acceptances) preclude someone from being entitled to weakly accept $A$, then they are committed to reject $A$ (and hence accept its negation):
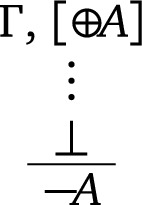


We can now return to the purported reduction of committive consequence to the bilateral interpretation of implication. Multilateralism cannot help with the problem that the reduction, even if successful, only works for single conclusions. I take this to speak in favour of natural deduction systems or single-conclusion sequent calculi. Nonetheless, the discussion should make it clear that multilateralism does help with the problem that the reduction did not work even in the case of single conclusions. For although PIA and PIR are not extensionally adequate, their counterpart formulated in terms of strong implication and strong incompatibility are:

[Pragmatic Implicit Acceptance* (PIA*)]

Any set of commitments that precludes entitlement to weakly reject $A$ implicitly commits one to accept $A$.

[Pragmatic Implicit Rejection* (PIR*)]

Any set of commitments that precludes entitlement to weakly accept $A$ implicitly commits one to accept $A$.

## Conclusion

I have argued that Hlobil and Brandom should become multilateralists.^11^ One outstanding issue is what the resulting logic would be if they did so using the many-sequent strategy. My conjecture is that in the current setting this would fail to secure classical logic even when the problematic structural rules hold. What seems to be needed is to add rules codifying the conditions under which if $\Gamma$ implies $\Delta$, then it also strongly implies it. In Incurvati and Schlöder 2023b, we take these conditions to be those in which the derivation preserves not only commitment but also evidence (a notion akin to Hlobil and Brandom’s entailment). It would be interesting to explore to what extent this approach could be successfully implemented in Hlobil and Brandom’s system.

I have not been able to cover many of the important topics discussed in RLLR, such as non-monotonicity and implication-space semantics. I hope to do so in future work. For now, let me conclude by reiterating my earlier statement that Hlobil and Brandom’s book is a major contribution to inferentialism. It will be a reference point for work on the topic for the years to come.

## Footnotes

1 Strictly speaking, this is the definition of permissive consequence Brandom adopted in later work (e.g., Brandom 2008).

2 Hlobil and Brandom also subscribe to a formulation in terms of speech acts (see, e.g., p. 180), but they mostly make use of the formulation in terms of attitudes. I will therefore stick to the latter for simplicity, but nothing hinges on this choice for the purposes of this paper.

3 Similar complaints have been raised by Rumfitt (2008) and Simonelli (forthcoming).

4 Simonelli (forthcoming) has recently pressed this worry in the context of the bilateral interpretation of the sequent calculus, but the worry is a familiar one for commitment-based accounts of meaning. In particular, it is what motivates the special account of disjunction in Simon Blackburn’s (1988) commitment semantics. See also Sonderholm 2005; Elstein 2007 for discussion.

5 Rea Golan (forthcoming), like Hlobil, Brandom and Simonelli a member of the Research On Logical Expressivism group, has argued that certain occurrences of sets of sentences on the right-hand side of a sequent can be read as unmade assumptions. I do not have space to discuss this interesting proposal. Suffices here to note that (as Golan himself observes) the proposal does not account for the right-disjunction rule adopted by Hlobil and Brandom (p. 109).

6 Our terminology differs from Hlobil and Brandom’s. We use *assertion* and *rejection* to refer to the speech acts expressing the attitudes we call *belief* and *disbelief*. For simplicity of exposition, I will stick to Hlobil and Brandom’s terminology here.

7 I say ‘*main* target’ because Brandom (1994, p. 115) already uses preclusion of entailment to characterize another notion of consequence, namely incompatibility-entailment, where $A$ incompatibly-entails $B$ just in case everything incompatible with $B$ is incompatible with $A$.

8 The two approaches can be shown to be inferentially equivalent (Francez, 2025), but this is compatible with their being meta-inferential or philosophical considerations for favouring one over the other.

9 Failures of reductio are not limited to the epistemic modal case, but also occur, for instance, in the case of probability operators (Incurvati and Schlöder 2023b: Ch. 9), truth (Incurvati & Schlöder, 2023a) and classes (Incurvati, 2025).

10 It is also possible to describe scenarios involving the use of epistemic modals that put pressure on Hlobil and Brandom’s Deduction-Detachment Condition on Conditionals, which states that a set of sentences $\Gamma$ implies $A \rightarrow B$ just in case $\Gamma$ and $A$ jointly imply $B$. However, these scenarios are more complex and admittedly more controversial, so I will not consider them here for reasons of space. It should also be noted that failures of conditional proof turn out to be crucial to defend committive consequence from a number of collapse arguments, as Simonelli and I show in ongoing work.

11 Incidentally, this is also the conclusion I reach in Incurvati forthcoming by focusing on issues about the function of logical vocabulary.
